# Revisiting antithrombotic therapeutics; sculptin, a novel specific, competitive, reversible, scissile and tight binding inhibitor of thrombin

**DOI:** 10.1038/s41598-017-01486-w

**Published:** 2017-05-03

**Authors:** Asif Iqbal, Mauricio Barbugiani Goldfeder, Rafael Marques-Porto, Huma Asif, Jean Gabriel de Souza, Fernanda Faria, Ana Marisa Chudzinski-Tavassi

**Affiliations:** 1 Laboratory of Biochemistry and Biophysics, Butantan Institute, Sao Paulo, SP Brazil; 2 Centre of Excellence in New Target Discovery (CENTD), Butantan Institute, São Paulo, SP Brazil; 3 Laboratory of Gene Expression in Eukaryotes, Butantan Institute, São Paulo, SP Brazil

## Abstract

Thrombin is a multifunctional enzyme with a key role in the coagulation cascade. Its functional modulation can culminate into normal blood coagulation or thrombosis. Thus, the identification of novel potent inhibitors of thrombin are of immense importance. Sculptin is the first specific thrombin inhibitor identified in the transcriptomics analysis of tick’s salivary glands. It consists of 168 residues having four similar repeats and evolutionary diverged from hirudin. Sculptin is a competitive, specific and reversible inhibitor of thrombin with a K_i_ of 18.3 ± 1.9 pM (*k*_on_ 4.04 ± 0.03 × 10^7^ M^−1^ s^−1^ and *k*_off_ 0.65 ± 0.04 × 10^−3^ s^−1^). It is slowly consumed by thrombin eventually losing its activity. Contrary, sculptin is hydrolyzed by factor Xa and each polypeptide fragment is able to inhibit thrombin independently. A single domain of sculptin alone retains ~45% of inhibitory activity, which could bind thrombin in a bivalent fashion. The formation of a small turn/helical-like structure by active site binding residues of sculptin might have made it a more potent thrombin inhibitor. In addition, sculptin prolongs global coagulation parameters. In conclusion, sculptin and its independent domain(s) have strong potential to become novel antithrombotic therapeutics.

## Introduction

Blood coagulation is a dynamic process, which involves a cascade of proenzymes leading to the activation of downstream enzymes^1–4^. In normal conditions, it results in haemostasis. This thrombo-hemorrhagic balance is critically maintained in the body by a complex and delicate mechanism. However, its disorder can potentially result in hemorrhage or thrombosis^2, 4^. Thrombin, a 37 kDa heterodimer, is a central enzyme in the coagulation cascade. Thrombin is a multifunctional enzyme, which functions as a pro-coagulant by cleaving of fibrinogen, activating coagulation factors (V, VIII, XI and XIII) and inducing platelet aggregation^4^. On the other hand, thrombin may function as an anticoagulant by binding to thrombomodulin and activating protein C. In addition, it plays a vital role in arterial and venous thrombosis, disseminated intravascular coagulation (DIC), cancer, inflammatory brain diseases, wound healing and atherosclerosis^3, 5, 6^. To overcome its detrimental effects, thrombin can be inhibited either directly or indirectly by blocking one or two of its three domains, i.e. active site and exosite 1 and 2^7^. Traditionally, unfractionated heparin (UFH) and low-molecular weight heparin (LMWH) were used as anticoagulants to inhibit thrombin in an indirect way by simultaneously binding to antithrombin and exosite 2 of thrombin^6, 8, 9^. However, heparin (UFH and LMWH) produces a fibrin-thrombin bridge and further thrombus formation, and may cause heparin-induced thrombocytopenia^5, 10^. Direct thrombin inhibitors (DTIs) constitute a group of anticoagulants that do not require cofactors, which bind directly to thrombin active site and obstruct its activity. DTIs have an advantage over indirect inhibitors because DTIs are more predictable anticoagulants as they lack an anti-platelet effect and do not lead to immune-mediated thrombocytopenia^2^. Several DTIs including recombinant hirudin and their hirulogs, are approved for use as anticoagulants^5^. Hirudin, a 65 amino acids peptide (7 kDa) is a direct thrombin inhibitor that was first isolated from the saliva of the medicinal leech *Hirudo medicinalis* ^11^. Later, a recombinant form of hirudin was produced that differs from native hirudin at Tyr^63^ residue, which is not sulfated^11, 12^. This difference marginally reduced the activity of recombinant hirudin. However, inhibition of thrombin by recombinant hirudin is irreversible and generates anti-hirudin antibodies, which culminates in accumulation of the drug. Currently, no antidote is available to revert the consequences of recombinant hirudin^2^. In addition, several synthetic hirulogs were developed and tested for their thrombin inhibitory activity but were nearly 800 times weaker inhibitors than recombinant hirudin^13^. Among all hirulogs, bivalirudin is a FDA approved anticoagulant which is a direct thrombin inhibitor but with a short half-life^2, 13^. Almost all of those anticoagulants are associated with side effects such as the formation of irreversible hirudin-thrombin complex, short half-life of the hirulogs and their dosage needs to be strictly monitored^2, 5^. Most of these thrombin inhibitors are from leeches and have been extensively investigated. On the other hand, specific thrombin inhibitors from ticks have been totally overlooked, albeit Kunitz-type inhibitors have been investigated^14–17^. Here, we reported the mechanism of the antithrombotic properties of sculptin, a newly identified molecule in the transcriptomics profile of the salivary glands from the tick *Amblyomma cajennense* (currently *Amblyomma sculptum* ^18^), which showed strong antithrombotic properties. Sculptin selectively and reversibly inhibited thrombin in a competitive manner. It was slowly cleaved by thrombin and factor Xa. Based on mass spectrometry and Edman analysis, we propose the thrombin active site binding sequence, which has only few conserved residues compared to classical hirudin from the medicinal leech but present sthe same potency. Sculptin has strong potential to become an antithrombotic drug and it could replace the classical hirudin and its analogs.

## Results

### Sequence analysis and phylogeny of sculptin

**Figure 1:**
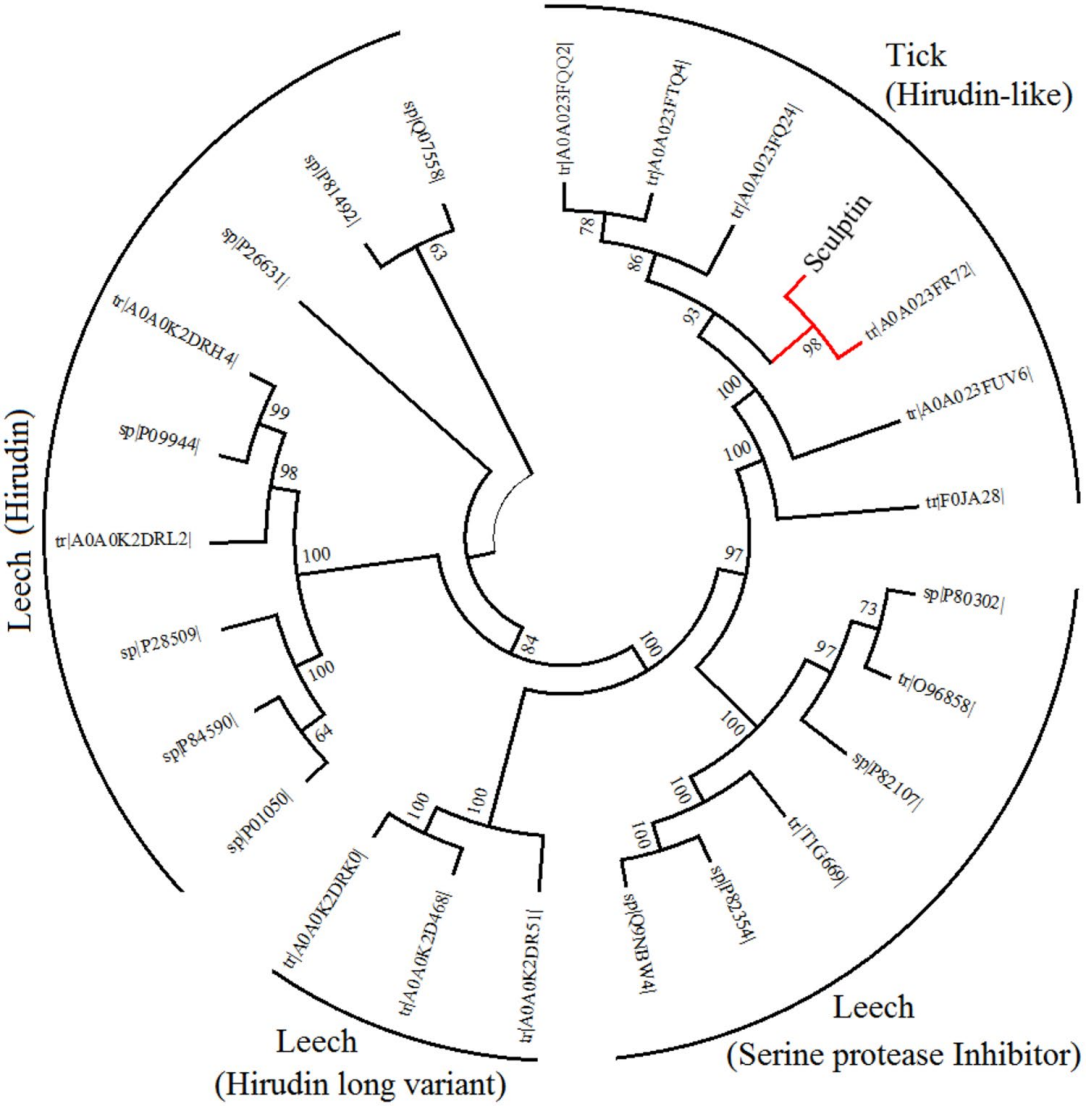
Phylogenetic analysis of Sculptin. Protein sequences of thrombin inhibitors from ticks and leeches were retrieved from Swiss-Prot/TrEMBL database (www.uniprot.org) and the phylogenetic profile was inferred using the Neighbor-Joining method using MEGA 7.0. The bootstrap consensus were 100 and cut-off value for condensed tree was 60% collapsed bootstrap replicates (see, experimental procedures). The accession number of each sequence is given and query position is highlighted in red. A single domain of sculptin was taken in to account during phylogeny construction.

Sculptin sequence was identified in the transcriptomics profile of the salivary glands from *Amblyomma cajennense* ^19^ (currently *Amblyomma sculptum* ^18^). Sculptin, a 168 amino acids polypeptide consists of a signal peptide, and four exactly similar repeats of 34 amino acids (Fig. S1A). The multiple alignment with classical hirudin from medicinal leech showed only few similarities and even the residues binding to thrombin active site were not conserved. The phylogenetic analysis of a single repeat domain of sculptin with other serine protease inhibitors suggested that it shares a common ancestor with hirudin variants from leeches but diverged in time of evolution. In fact, in the evolution tree, sculptin was closer to serine protease inhibitors of the antistasin family i.e. hirustasin, guamerin, bdellastasin, theromin and therostasin than classical hirudin from leech. As expected, sculptin belonged to same family of the hirudin like sequences from tick (Fig. 1).

### Purification of recombinant sculptin

**Figure 2:**
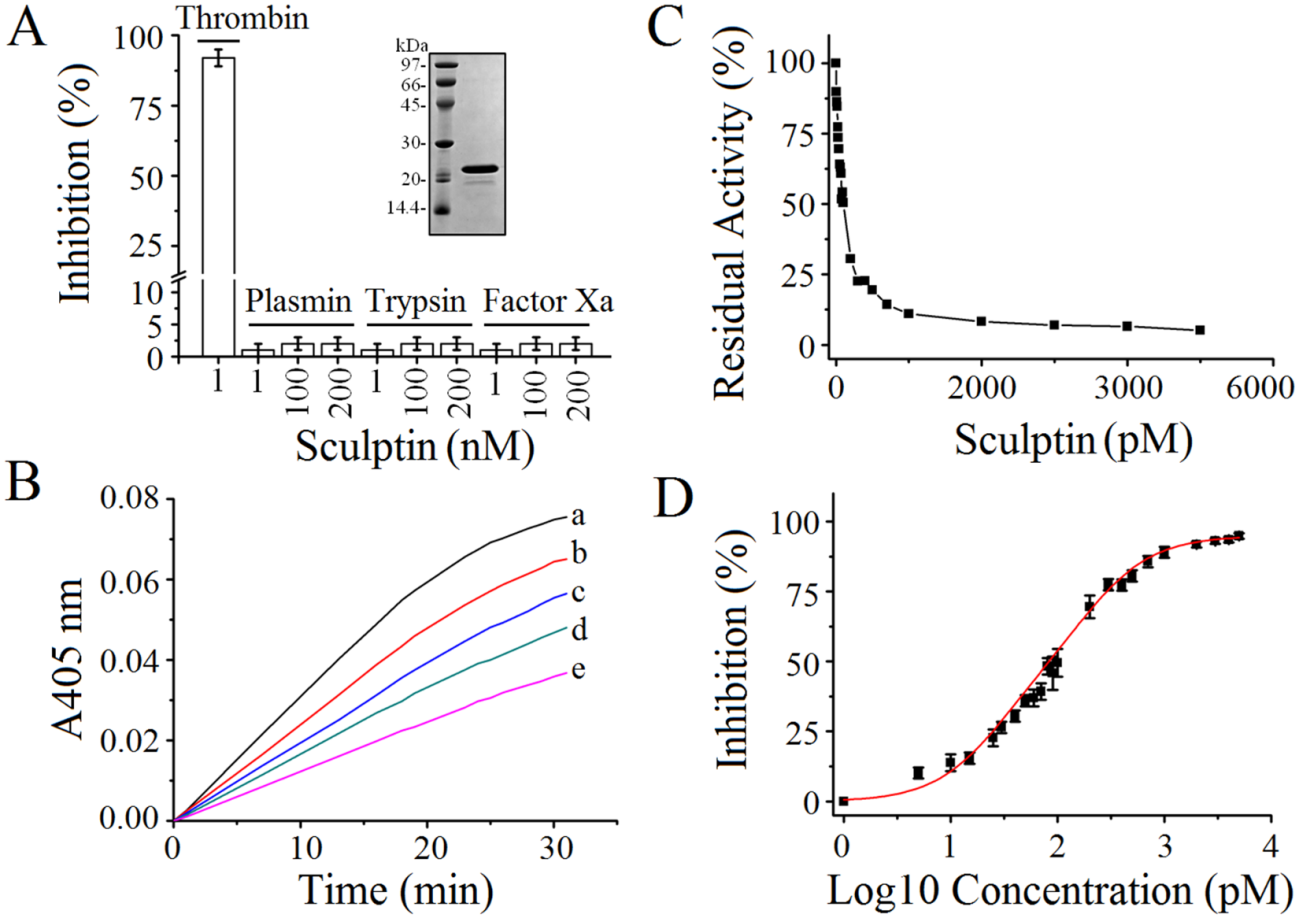
Sculptin specificity for thrombin and its dose-dependency and IC_50_ value for thrombin inhibition. (**A**) The inhibition of serine proteases by sculptin. Serine protease (100 pM; thrombin, plasmin, trypsin or factor Xa) was incubated with sculptin (1, 100 and 200 nM) in 50 mM phosphate buffer containing 150 mM NaCl and 0.1% PEG 6000, pH 7.4 for 6 h at 37 °C. After addition of the corresponding chromogenic substrate to the reaction mixture, its hydrolysis was monitored at 405 nm. For factor Xa activity, the buffer contained 50 µM phosphatidylserine and phosphatidylcholine. The inset in (**A**) shows the SDSPAGE of purified recombinant sculptin, which was used in the experiments. (**B**) Typical curves for hydrolysis of S-2238 chromogenic substrate (15 µM) by 0.1 nM thrombin in the absence (trace a) or presence of sculptin (trace b, 15 pM; trace c, 30 pM; trace d, 60 pM and trace e, 100 pM) in 50 mM phosphate buffer containing 150 mM NaCl and 0.1% PEG 6000, pH 7.4 at 37 °C. (**C**) Residual activity of thrombin in presence of increasing concentration of sculptin. (**D**) Dose-response curve for thrombin inhibition by sculptin. The percentage of thrombin inhibition was plotted versus the log of sculptin concentration. The experimental condition of (**C**) and (**D**) is the same as in (**B**). The results shown in (**C**) and (**D**) correspond to the mean ± standard deviation values acquired in three independent experiments.

The synthetic construct of sculptin without signal peptide and with a C-terminal polyhistidine-tag was cloned into the pET28a expression vector. Recombinant sculptin well expressed and was mostly present in the soluble fraction (Fig. S2). Sculptin was purified by conventional affinity and ion exchange chromatography (Fig. S2). Mass spectrometry analysis indicated a 16990.90 Da mass (Fig. S3A) for the purified sculptin, and on SDS-PAGE, it runs just above the 20 kDa marker band (Fig. S3B). The purified recombinant sculptin was used for further experiments (inset in Fig. 2A).

### Sculptin is a specific inhibitor of thrombin

As sculptin was closed to the antistasin family inhibitors in an evolution tree, the first experiment we conducted was aimed at testing of serine proteases inhibition capacity of sculptin. For this purpose, we selected thrombin, trypsin, plasmin and factor Xa. The hydrolysis of a chromogenic substrate by serine proteases in presence and absence of sculptin was monitored spectrophotometrically. At the concentration of 1 nM, sculptin decreased the residual activity of thrombin by around 97% (Fig. 2A). On the other hand, sculptin (1, 100, 200 nM) did not inhibit factor Xa, trypsin and plasmin (Fig. 2A).

### Inhibition of residual activity of thrombin by sculptin and IC_50_ value calculation

Thrombin was the only enzyme inhibited by sculptin. We further analyzed the inhibition of thrombin with increasing concentrations of the inhibitor. The data showed that at increasing the concentration, sculptin decreased the residual activity of thrombin (Fig. 2B–D). The plot of percent inhibition versus log of concentration was fitted into dose-response function of equation 1 and the IC_50_ value of 86.6 ± 1.9 pM was calculated (Fig. 2D).

### Kinetics of thrombin inhibition by sculptin

**Figure 3:**
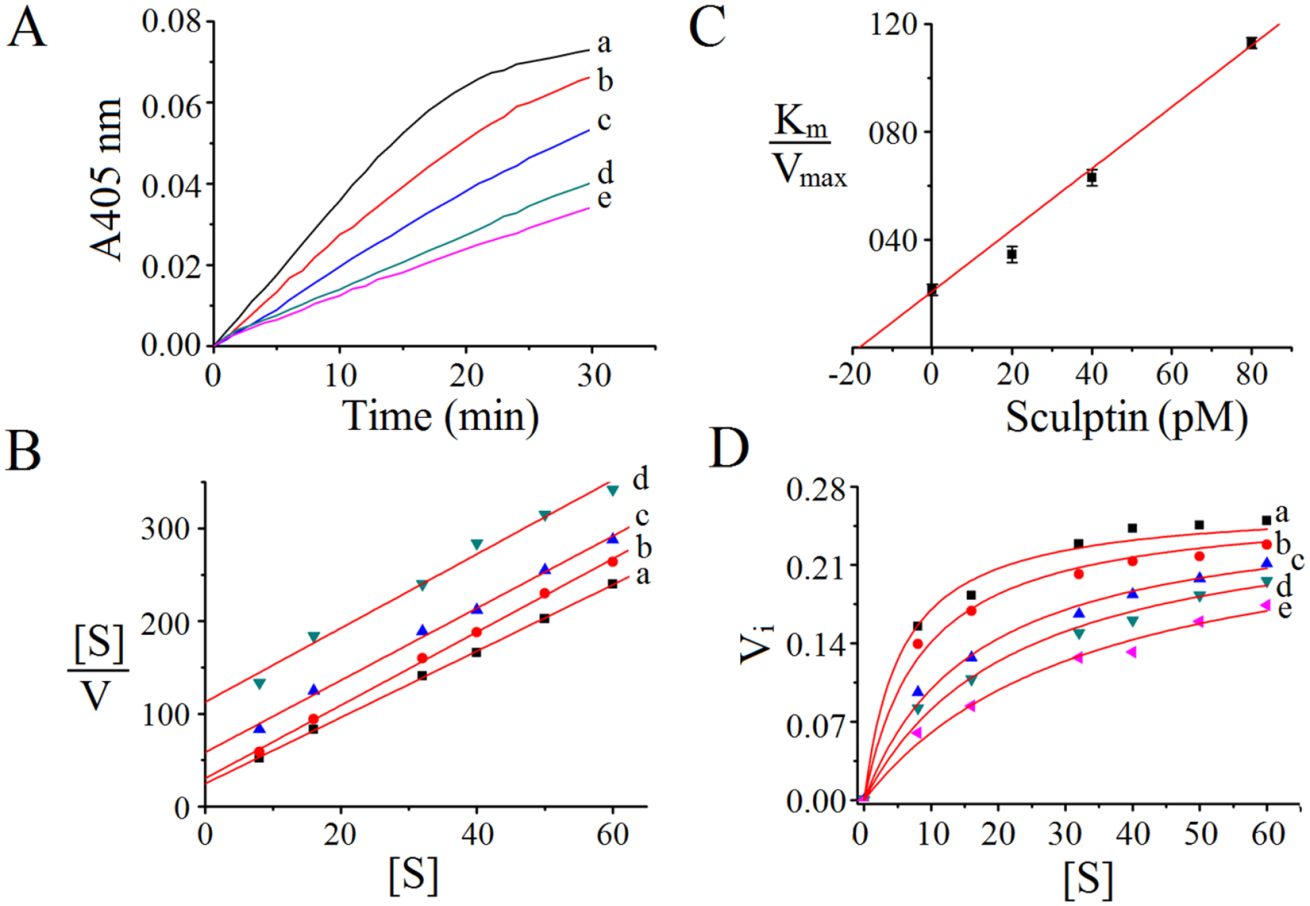
Kinetics of inhibition of thrombin by sculptin. (**A**) Typical progress curves for hydrolysis of S-2238 chromogenic substrate by 0.1 nM thrombin in absence (trace a) and presence of sculptin (trace b, 20 pM; trace c, 40 pM; trace d, 60 pM and trace e, 80 pM) in 50 mM phosphate buffer containing 150 mM NaCl and 0.1% PEG 6000, pH 7.4 at 37 °C. Reactions were started with the addition of thrombin to the mixture containing sculptin and S-2238. (**B**) The Hanes-Woolf plot for the inhibition of thrombin by sculptin (trace a, 0 pM; trace b, 20 pM; trace c, 40 pM and trace d, 80 pM). The substrate concentrations divided by its corresponding initial velocities of thrombin inhibition by sculptin were plotted vs sculptin concentration using Eq. (2). (**C**) The intercept of the (**B**) was plotted versus respective concentration to obtain Ki. (**D**) The nonlinear regression for competitive inhibition using Eqs (3) and (4). The initial velocity of thrombin inhibition in absence (trace a) and presence of sculptin (trace b, 20 pM; trace c, 40 pM; trace d, 60 pM and trace e, 80 pM) at different substrate concentrations. The experimental condition of (**B**,**C**) and (**D**) is the same as (**A**). The data in (**B**,**C**) and (**D**) correspond to the mean ± standard deviation values acquired in five independent experiments.

In order to evaluate the type of inhibition exercised by sculptin on thrombin, we determined the kinetic parameters of hydrolysis of the chromogenic substrate S-2238 by thrombin in the presence of sculptin. For this purpose, several assays were conducted using (i) a fixed substrate concentration and increasing concentrations of sculptin; and (ii) a fixed sculptin concentration and increasing concentrations of S-2238. Typical hydrolysis curves of S-2238 by thrombin are given in Fig. 3A. The data were fitted into the Hanes-Woolf plot, which confirmed the competitive nature of sculptin (Fig. 3B). The K_i_ obtained for thrombin inhibition by sculptin was 18.3 ± 1.9 pM (Fig. 3C) and it was further confirmed by fitting the data into non-linear regression for competitive enzyme inhibition using equations 3 and 4 (Fig. 3D).

### Binding kinetics of sculptin to thrombin

**Figure 4:**
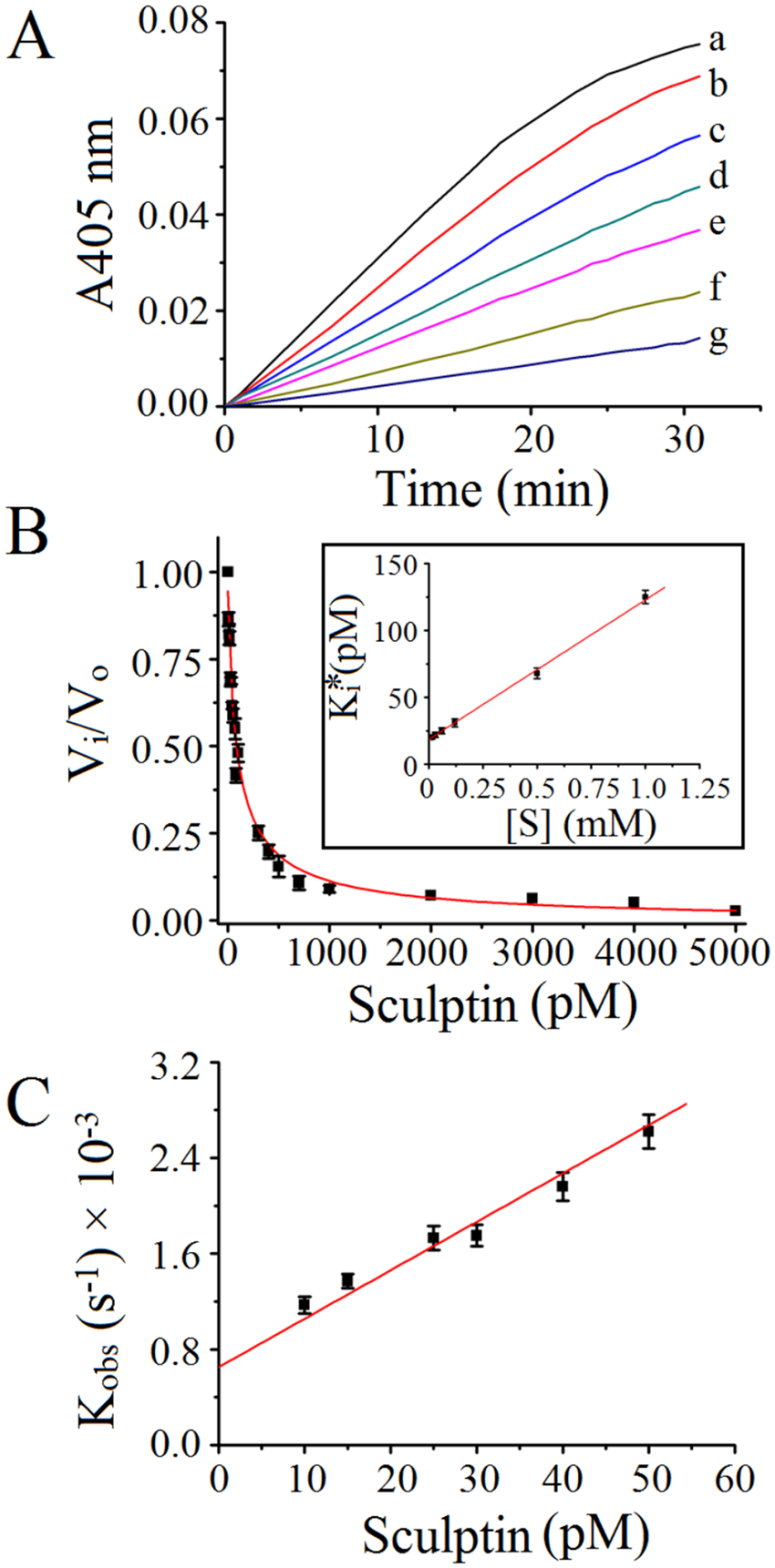
Relationship between the apparent first-order rate and the concentration of tight binding inhibitor sculptin. (**A**) Typical progress curves for hydrolysis of 15 µM S-2238 chromogenic substrate by 0.1 nM thrombin in absence (trace a) and presence of sculptin (trace b, 10 pM; trace c, 30 pM; trace d, 70 pM; trace e, 100 pM; trace f, 200 pM and trace g, 500 pM) in 50 mM phosphate buffer containing 150 mM NaCl and 0.1% PEG 6000, pH 7.4 at 37 °C. Reactions were started with addition of thrombin to the mixture containing sculptin and S-2238. (**B**) Steady-state velocity of thrombin with respect to sculptin concentration. The inset shows the determination of the apparent dissociation constant, Ki*, from steady state velocities. The data was fitted into a linear regression to obtain Ki. (**C**) Calculation of the dissociation constant from k_obs_. Progress curves were produced with 15 µM S-2238, 0–50 pM sculptin, and 100 pM thrombin. The apparent first-order rate constant was calculated using a nonlinear regression fit, where the intercept and slope is k_on_ and k_off_ respectively. The experimental condition of (**B**) and (**C**) is the same as (**A**). The data in (**B**) and (**C**) correspond to the mean ± standard deviation values acquired in five independent experiments.

For binding kinetics, the premixed substrate and sculptin concentrations were added to the reaction mixtures already containing thrombin (see experimental procedure). The inhibition traces are straight and separated lines right from the start of the reaction, thus suggesting fast and tight binding between sculptin and thrombin (Fig. 4A). In addition, fractional velocities were plotted versus inhibitor concentrations using Morrison’s tight binding equation 5 and the data best fit in the equation (Fig. 4B). K_i_ of 19.5 ± 3.5 pM was calculated by Morrison’s tight binding equation, which was similar to that determined by non-linear regression for competitive enzyme inhibition. Furthermore, k_obs_ calculated using equation 6 was plotted versus sculptin concentration. From the plot, we calculated *k*_on_ and *k*_off_, which was 4.04 ± 0.03 × 10^7^ M^−1^ s^−1^ and 0.65 ± 0.04 × 10^−3^ s^−1^ respectively (Fig. 4C). The inhibition constant (K_i_) of 16.1 ± 1.4 pM was calculated using equation 7.

### Sculptin degradation by serine proteases

**Figure 5:**
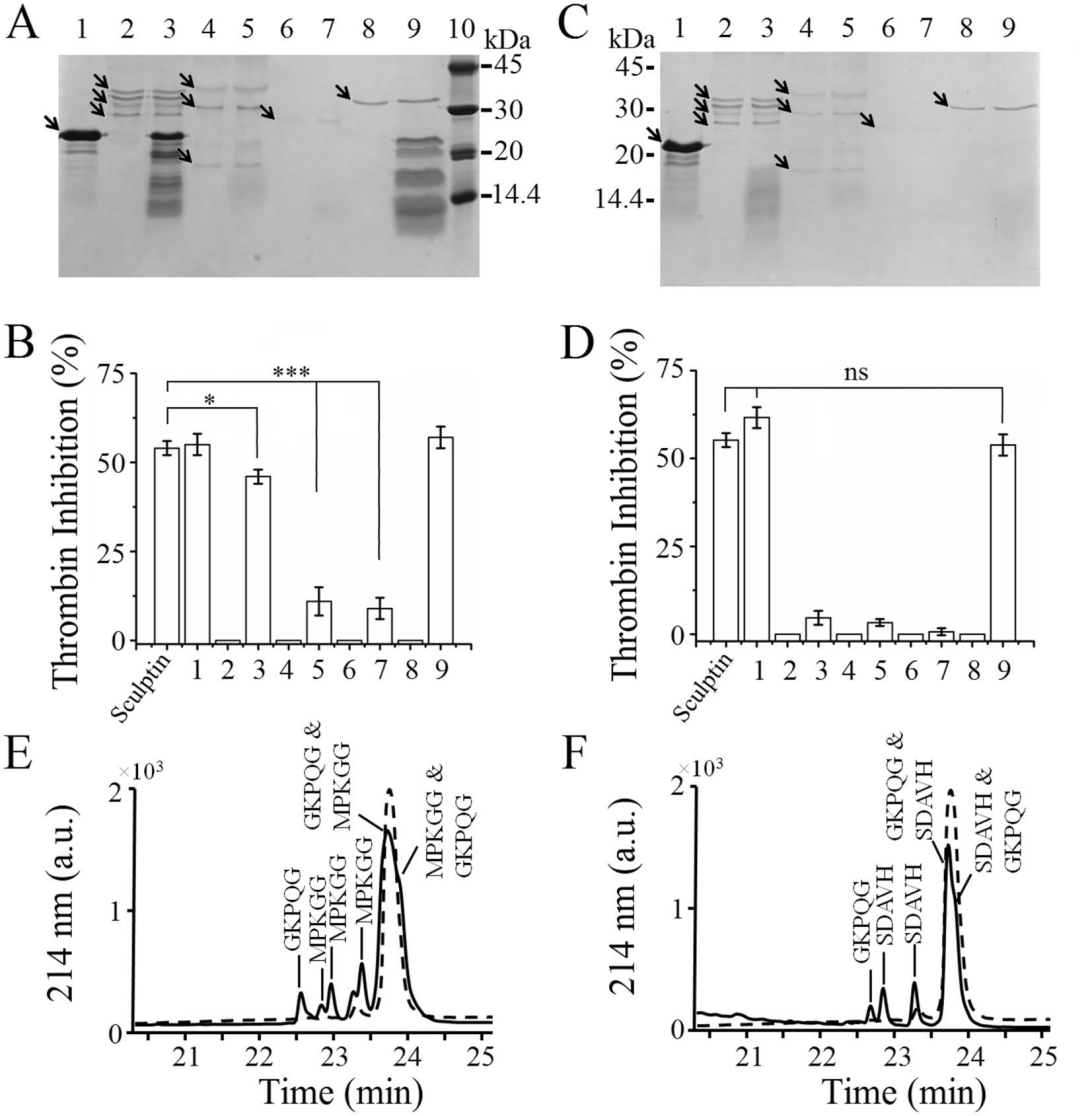
Degradation of sculptin by serine proteases and its thrombin inhibition activity. Sculptin (10 µM) was incubated without or with 1 µM of serine protease (thrombin, plasmin, trypsin or factor Xa) in 50 mM phosphate buffer containing 150 mM NaCl and 0.1% PEG 6000, pH 7.4 for 4, 6, 7 or 18 h at 37 °C. The reaction mixtures (20 µl) were separated by SDS-PAGE. (**A**) SDS-PAGE (15%) of sculptin hydrolysis by serine proteases after 6 h incubation. (**B**) Percent thrombin inhibition by sculptin after 6 h incubation with serine proteases (see experimental procedures). (**C**) SDS-PAGE (15%) of sculptin hydrolysis by serine proteases after 18 h incubation. (**D**) Percent thrombin inhibition by sculptin after 18 h incubation with serine protease (see experimental procedures). The numbering of (**B**) and (**D**) correspond to the numbering of (**A**) and (**C**) respectively and sculptin control is represented by CTRL. Sculptin (lane 1); thrombin (lane 2) and thrombin with sculptin (lane 3); plasmin (lane 4) and plasmin with sculptin (lane 5); trypsin (lane 6) and trypsin with sculptin (lane 7); factor Xa (lane 8) and factor Xa with sculptin (lane 9) and protein marker (lane 10; in A). (**E**) Identification of thrombin cleavage sites in sculptin after 7 h of incubation. (**F**) Identification of factor Xa cleavage sites in sculptin after 4 h of incubation. Thrombin and Factor Xa cleavage sites in sculptin sequence are given in Figs S1 and S2 respectively. The experimental procedure for (**C**–**E**) and (**F**) was same for (**A**) and (**B**) except incubation time and type of serine protease used. The results shown in (**B**) and (**D**) correspond to the mean ± standard deviation values acquired in three independent experiments; ns nonsignificant, ***p < 0.001 and *p < 0.05.

Next, we determined whether serine proteases such as thrombin, plasmin, factor Xa and trypsin hydrolyze sculptin. For this purpose, sculptin (10 µM) was incubated without or with 1 µM of serine protease (thrombin, plasmin, trypsin or factor Xa) in 50 mM phosphate buffer containing 150 mM NaCl and 0.1% PEG, pH 7.4 for 6 h or 18 h at 37 °C. SDS-PAGE of the reaction mixture after 6 h of incubation showed that compared to the sculptin control band, the intensity of the 20-kDa band (corresponding to undigested sculptin) decreased and lower molecular weight bands appeared in thrombin-incubated sculptin (Fig. 5A). On the other hand, after 6 h of incubation, the 20-kDa band completely disappeared in both plasmin- and trypsin-incubated sculptin (Fig. 5A). Likewise, after the same time of incubation, factor Xa also converted sculptin to fragments (Fig. 5A). For thrombin inhibition assay, the same samples were diluted 100 thousand times for a final sculptin concentration of 100 pM and the reaction mixtures were further supplemented with 100 pM thrombin. Upon addition of the chromogenic substrate S-2238, its hydrolysis by thrombin was monitored spectrophotometrically. The data showed that sculptin incubated without serine protease for 6 h inhibited thrombin similar to the control (control is fresh 100 pM sculptin; Fig. 5B). On the other hand, sculptin incubated with thrombin had its inhibitory activity decreased by 20% and sculptin incubated for 6 h with plasmin or trypsin had its inhibitory activity decreased by 80% (Fig. 5B). In addition, we also incubated sculptin with thrombin and monitored the deactivation of sculptin over the time (Fig. S4). A significant reduction in the activity of sculptin was observed after 4 h incubation (Fig. S4A). A second order rate constant for sculptin degradation/deactivation by thrombin was roughly 3.4 ± 0.7 × 10^−8^ M^−1^ h^−1^ (Fig. S4B). Interestingly, sculptin digested with factor Xa retained its thrombin inhibition activity (Fig. 5B). Next, we examined sculptin incubated with serine proteases for 18 h. The 20-kDa band that corresponds to sculptin monomer was completely disappeared from the reaction mixtures (Fig. 5C). Likewise, for the thrombin inhibition assay, the samples were diluted 100 thousand times and were further supplemented with 100 pM thrombin. As expected, sculptin incubated without serine protease for 18 h inhibited thrombin similar to the fresh sculptin control (Fig. 5D). However, sculptin incubated with thrombin, plasmin and trypsin did not inhibit thrombin (Fig. 5D). Interestingly, sculptin incubated with factor Xa still inhibited thrombin activity (Fig. 5D).

### N-terminal sequencing of sculptin hydrolyzed by thrombin

As we discussed above, thrombin degrades sculptin. Henceforth, our next step was to determine thrombin cleavage sites in the sequence of sculptin. For this purpose, we incubated sculptin with thrombin for 7 h and the peptides generated during hydrolysis were separated by reversed phase chromatography. The individual peaks were collected and were subjected to Edman N-terminal sequencing. The residues sequenced for the first peak were GKPQG, which are the first five residues of sculptin (Fig. 5E). The residues sequenced for the next three peaks (2^nd^, 3^rd^, and 4^th^) were MPKGG, which are basically the N-terminal residues of sculptin peptides generated by thrombin (Fig. 5E). The last peak (5^th^) with a retention time equal to the control’s was sequenced to have both MPKGG and GKPQG residues at the N-terminal suggesting that this peak harbors both intact and partially degraded sculptin (Fig. 5E). The fractions were also subjected to mass spectrometry, which were in agreement with Edman sequencing data (Table S1). The theoretical and experimental masses of the peptides are listed in Table S1.

### N-terminal sequencing of sculptin hydrolyzed by factor Xa

We also determined factor Xa cleavage sites in sculptin. The peptides generated by incubation of sculptin with factor Xa for 4 h were separated by reversed phase chromatography. Edman sequencing showed that the N-terminal residues for the first peak were GKPQG, which are the first five residues of sculptin (Fig. 5F). The residues sequenced for the next two peaks (2^nd^ and 3^rd^) were SDAVH, which are actually the N-terminal residues of sculptin peptides generated by factor Xa (Fig. 5F). The last peak (4^th^) with retention time equal to the control was sequenced to have both MPKGG and SDAVH N-terminal residues suggesting that this peak harbors both intact and partially degraded sculptin (Fig. 5F). Next, collected peaks were subjected to mass spectrometry, which was in agreement with Edman sequencing data (Table S2). The theoretical and experimental masses of the peptides are listed in Table S2.

### Factor Xa generated sculptin fragments retain thrombin inhibition activity

**Figure 6:**
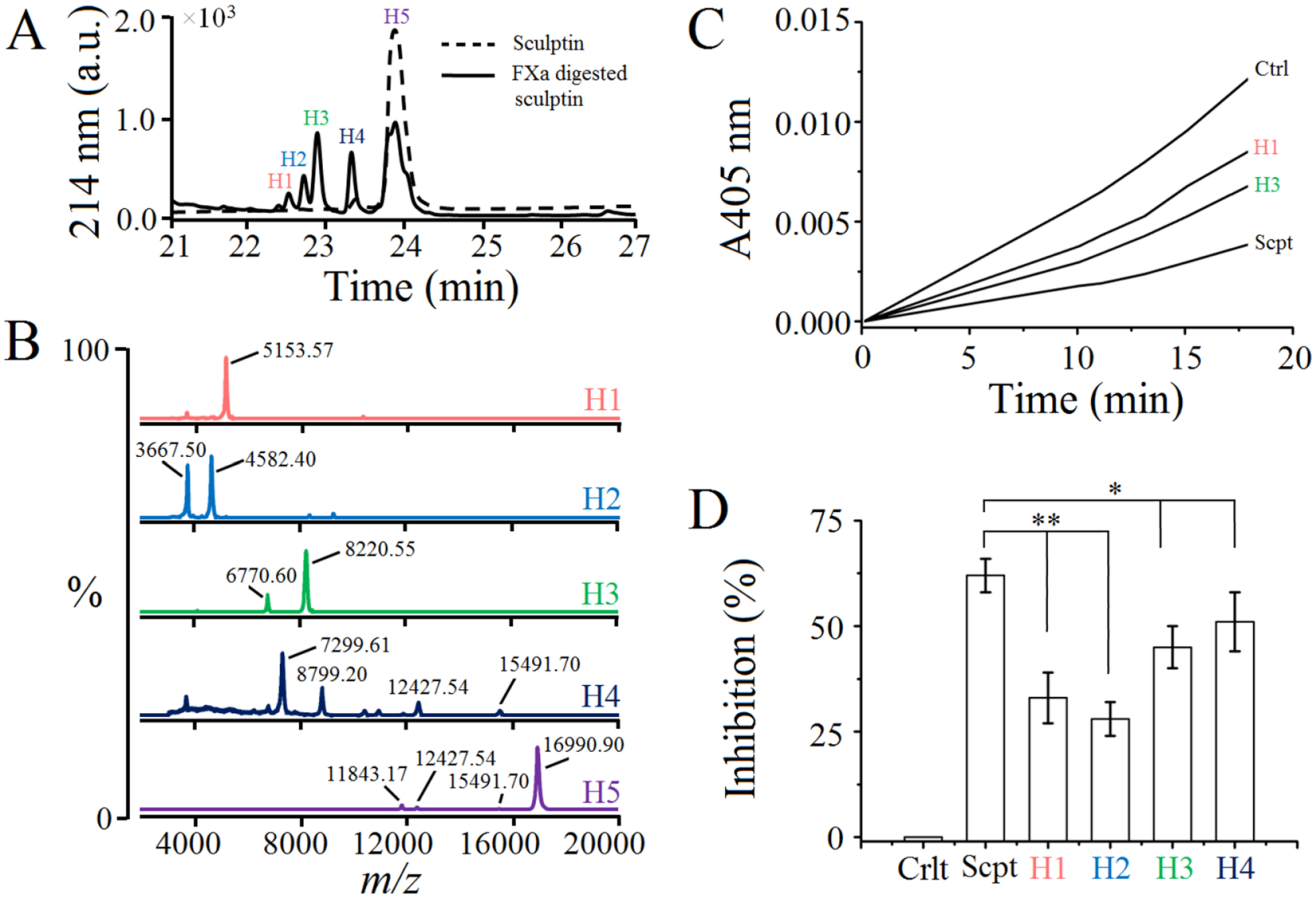
Thrombin inhibition activity of sculptin fragments generated by factor Xa. Sculptin (10 µM) was incubated without or with 1 µM of factor Xa in 50 mM phosphate buffer containing 150 mM NaCl, and 50 µM phosphatidylserine and phosphatidylcholine, pH 7.4 for 6 h at 37 °C. (**A**) The reaction mixtures were separated by reversed phase C-18 HPLC column. (**B**) The collected peaks (H1–H5) were subjected to MALDI-TOF MS and thrombin inhibition assay (see Table 1, for sequence of the corresponding peptides). (**C**) Typical progress curves for hydrolysis of 15 µM S-2238 chromogenic substrate by 0.1 nM thrombin in the absence (trace Ctrl) and presence of 100 pM of sculptin fragment (traces H1 and H3) or intact sculptin (trance Scpt) in 50 mM phosphate buffer containing 150 mM NaCl and 0.1% PEG 6000, pH 7.4 at 37 °C. (**D**) Percent thrombin inhibition by sculptin and its fragments. The reaction conditions of (**D**) are same as in (**C**). The results shown in (**D**) correspond to the mean ± standard deviation values acquired in three independent experiments; **p < 0.01 and *p < 0.05.

Furthermore, sculptin was incubated with factor Xa for 18 h and the resulting peptides were separated by reversed phase chromatography (Fig. 6A). The peaks (named as H1, H2, H3 H4 and H5) were collected and were subjected to MALDI-TOF mass spectrometry (Fig. 6B). According to the mass spectrometry analysis H1 corresponds to the average mass of 5153.57 Da, H2 corresponds to the average mass of 3667.50 Da and 4582.40 Da and H3 corresponds to the average mass of 8220.55 Da and 6770.60 Da. Similarly, H4 corresponds to the average mass of 7299.61 Da and 12427.54 Da and H5 corresponds to 16990.90, 12427.54 Da and 11843.17 Da (Fig. 6B, Table 1). In addition, the fractions (H1, H2, H3 and H4) were subjected to thrombin inhibition assay (Fig. 6C). The fractions H1, H2, H3 and H4 retained thrombin inhibition activity of approximately 50%, 45%, 70% and 80% respectively, of the intact non-hydrolyzed sculptin (Fig. 6D). Peak H5 were mostly of intact sculptin, so it was not considered for the thrombin inhibition assay.

**Table 1:** The fragments of sculptin generated by factor Xa.

Fragment number^a^	Fragment of sculptin^b^	Theoretical Average Mass	Calculated Average Mass^c^
H1^d^	GKPQGHPHDALEARSDAVHTAVPKMPKGGHGGFEPIPIDYDERALEAR	5156.74	5153.57
H2^d^	SDAVHTAVPKMPKGGHGGFEPIPIDYDERALEAR	3663.10	3667.50
	SDAVHTAVPKMPKGGHGGFEPIPIDYDERALHALEHHHHHH	4579.05	4582.40
H3^d^	SDAVHTAVPKMPKGGHGGFEPIPIDYDERALEARSDAVHTAVPKMPKGGHGGFEPIPIDYDERALHALEHHHHHH	8223.13	8220.55
	SDAVHTAVPKMPKGGHGGFEPIPIDYDERALEARSDAVHTAVPKMPKGGHGGFEPIPIDYDER	6765.55	6770.60
H4^d^	SDAVHTAVPKMPKGGHGGFEPIPIDYDERALEARSDAVHTAVPKMPKGGHGGFEPIPIDYDERALEAR	7306.17	7299.61
	GKPQGHPHDALEARSDAVHTAVPKMPKGGHGGFEPIPIDYDERALEARSDAVHTAVPKMPKGGLGGFEPIPIDYDERALEARSDAVHTAVPKMPKGGHGGFEPIPIDYDERALEAR	12420.90	12427.54

Sculptin (10 µM) was incubated without or with 1 µM of factor Xa in 50 mM phosphate buffer containing 150 mM NaCl and 50 µM PS/PC, pH 7.4 for 6 h at 37 °C. The reaction mixtures were separated by reverse phase C-18 HPLC column. The peaks were subjected to MALDI-TOF MS.

^a^Peak number of the HPLC chromatogram (Fig. 5A).

^b^Fragment of sculptin generated by factor Xa.

^c^The experimental average mass calculated by MALDI-TOF mass spectrometry (Fig. 5B).

^d^The fractions (HPLC peaks) were subjected to thrombin inhibition test (Fig. 5C,D).

### Comparative modeling and docking of sculptin

**Figure 7:**
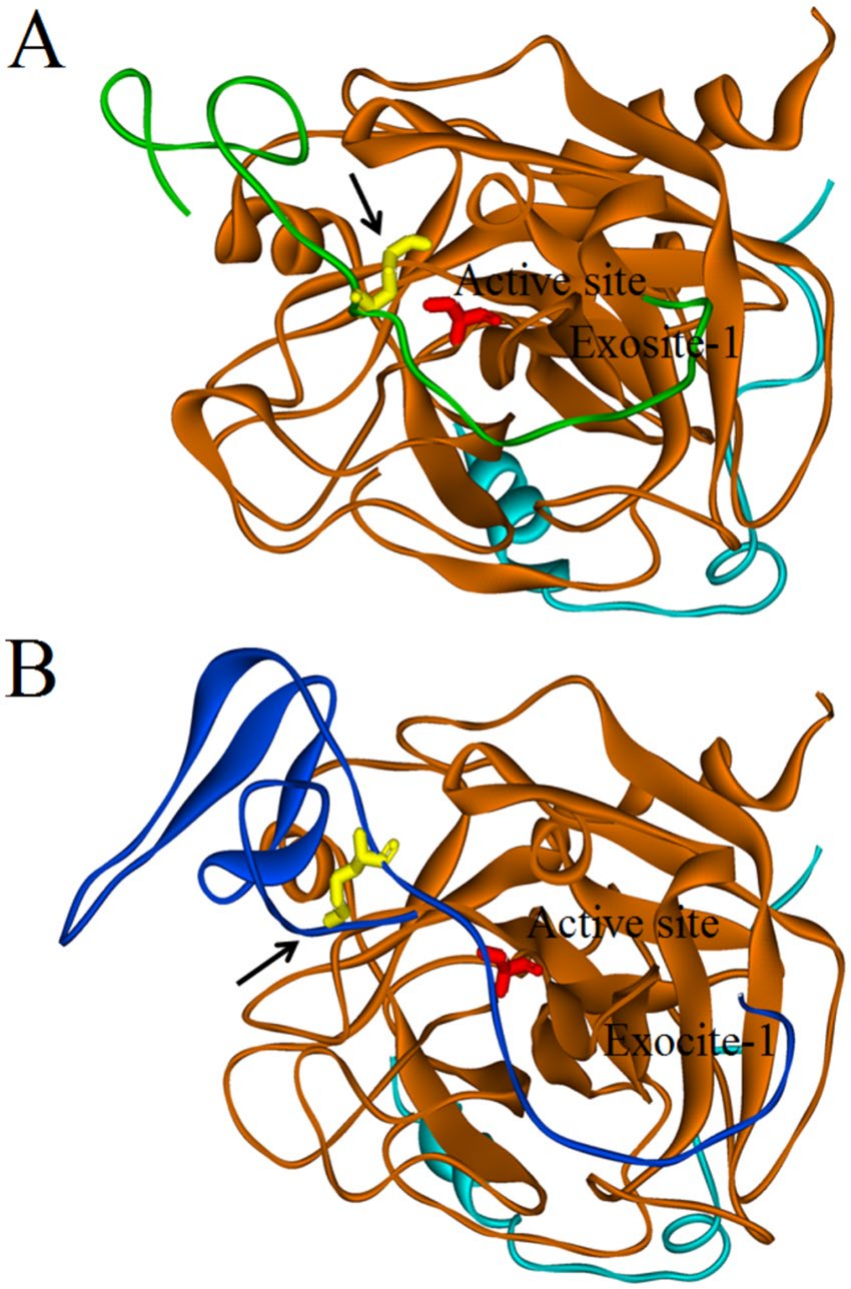
Cartoon (Solid ribbon) representation of comparative structure of sculptin (single domain) and crystal structure of hirudin bound to thrombin. The brown and cyan color represent the heavy and light chains of thrombin respectively. (**A**) Sculptin in green bound to thrombin. (**B**) Hirudin in blue bound to thrombin. Lys residue of the inhibitors is shown in yellow and Ser^195^ residue of the thrombin active site is presented in red. The arrow indicates the active site binding pocket of the thrombin occupied by Lys^23^ residue of sculptin (**B**) and by the N-terminal region of hirudin (**A**).

Fraction H1, which corresponds to a single domain of sculptin, retained approximately 45% inhibition activity. For this reason, we proposed a comparative docking model of the single domain of sculptin to thrombin using a crystal structure of the thrombin-hirudin complex. The comparative model of sculptin (single domain) docked to the thrombin active site and crystal structure of hirudin complex with thrombin are given in Fig. 7. The crystal structure of the hirudin-thrombin complex suggests that the N-terminal residues of hirudin resides in the active site pocket of thrombin, which is stabilized by several hydrogen bonds (Fig. 7B). On the other hand, the docking of a single domain of sculptin proposed that Lys^23^ occupies the active site pocket of thrombin instead of the N-terminal residues (Fig. 7A). In our model, Lys^23^ of sculptin was stabilized by a hydrogen bond with the main chain of Pro^96^ of thrombin (Figs 7A and S5A). Lys^23^ of sculptin is comparatively flexible due to the presence Met^24^ at P2 position (^22^PKM^24^ in sculptin) when compared to Lys^47^ residue of hirudin with Pro^48^ at P2 position (^46^PKP^48^ in hirudin). Furthermore, the predicted hydrogen bonds of sculptin (single domain) with thrombin were compared with the C-terminal domain of hirudin. There were three common hydrogen bonds formed by sculptin and by hirudin with thrombin residues i.e. Arg^35^, Lys^36^, and Glu^39^ (underlined; Fig. S5A,B). In addition, each inhibitor was making two uncommon hydrogen bonds with thrombin residues, i.e. Arg^75^ and Ty^r60^ with hirudin, and Pro^61^ and Glu^194^ with sculptin (Fig. S5A,B). The distance between Ser^195^ of thrombin and peptide bond at the P1 position was 16.1 Å for sculptin and 14.3 Å for hirudin. Furthermore, the residues at P1′, P1, P2 and P3 position of hirudin (^46^PKPQ^49^) and sculptin (^22^PKMP^22^) were compared and superimposed (Fig. S5C). RMSD value of 0.98 Å (main chain RMSD) and 2.96 Å (global RMSD) was calculated.

### The effect of sculptin on aPTT, PT and TT

Finally, PT, aPTT and TT were evaluated in plasma isolated from healthy human volunteers after incubation with sculptin for 3 min at 37 °C. The data showed that aPTT and PT were prolonged by sculptin in a concentration dependent manner (Fig. S6A and B). The maximum assay reading for aPTT was reached after 12 nM, whereas, for PT it was reached after 6 nM of sculptin (Fig. S6A and B). On the other hand, TT was prolonged by sculptin in the pico-molar range (Fig. S5C).

## Discussion

In the transcriptomics data of the salivary glands from *Amblyomma cajennense* tick, we have identified a novel specific thrombin inhibitor named sculptin, a 168 amino acids polypeptide consisting of a signal peptide and four identical repeats of 34 amino acids^19^. To the best of our knowledge, this is the mechanistic of the antithrombotic properties of the first thrombin inhibitor of tick origin. Our data showed that each repeated domain of sculptin resemble the C-terminal cysteine-free half of leech hirudin. It suggests that the more complex and cysteine rich N-terminal region of hirudin was deleted during the course of evolution in sculptin^13^. In addition, the multiplication or rapid expansion of sculptin domain repeats is supposed to have evolved through tandem exon duplications or internal tandem duplications^20^. It could explain how, sculptin is phylogenetically diverged from classical hirudin from leeches. Even though, sculptin is a specific thrombin inhibitor, surprisingly it is evolutionarily closer to antistasin-type serine protease inhibitors such as hirustasin, guamerin, bdellastasin, theromin and therostasin. These molecules are the inhibitors of kallikrein, trypsin, chymotrypsin, elastases, acrosin, thrombin and factor Xa^21, 22^. Those evolutionary changes to the sculptin sequence made it easier to express in bacteria as a soluble protein as compared to leech hirudin, which are expressed as inclusion bodies and need extra refolding steps during purification^13, 23^. From a biotechnological point of view, this is quite a valued advantage of sculptin over the classical hirudin and, importantly, the sequence divergence did not compromise sculptin’s affinity for thrombin. Sculptin followed competitive kinetics with an inhibition constant (K_i_) of 18.3 ± 1.9 pM. Its K_i_ is comparable to the recombinant hirudin from medicinal leech but lower than hirudin derivatives^11, 13, 24–27^ (Table S3). The values of *k*_on_ (4.04 ± 0.03 × 10^7^ M^−1^ s^−1^) and *k*_off_ (0.65 ± 0.04 × 10^−3^ s^−1^), which is quite comparable to that of hirudin (Table S4) suggest that the interaction of sculptin with thrombin is very fast and nearly irreversible. However, thrombin degrades sculptin in the process (roughly *k* = 3.4 ± 0.7 × 10^−8^ M^−1^ h^−1^), which makes the inhibition reversible and different from hirudin^11, 26, 27^. Therefore, the sculptin-thrombin complex would not requires an antidote for its clearance^2^. In contrast, the hirudin-thrombin complex is irreversible and does require an antidote for its clearance^2, 5, 11^. The properties of sculptin such as reversibility and consumption by thrombin are somewhat similar to bivalirudin and argatroban^24^. However, those molecules are approximately 100 and 2000 times, respectively, less effective than sculptin^24–27^. In addition, bivalirudin and argatroban have very short half-life compared to 8 h for sculptin and 1.3 h for hirudin^2, 11, 13, 24^. The extraordinary half-life for sculptin may be attributed to its repeated domains, which may have the ability to inhibit thrombin independently. Furthermore, Edman sequencing and mass spectrometry analyses of sculptin degraded by thrombin allowed us to identify A, V, P, K, M, P and K residues at P4, P3, P2, P1, P1′, P2′ and P3′ respectively that bind to the corresponding subside of thrombin active site. The identification of different fragments of sculptin with the same thrombin active site binding residues suggest that each 32 repeated domain may has the ability to bind and inhibit thrombin independently.

Moreover, sculptin did not inhibit plasmin, trypsin and factor Xa, and those enzymes efficiently degraded it. Thus, sculptin may have no effect on the normal fibrinolysis process and may not result in thrombosis associated to plasmin functional inhibition. Upon digestion with plasmin and trypsin, sculptin completely lost thrombin inhibition activity. On the other hand, the fragments of sculptin generated by factor Xa are large compared to those produced by plasmin. Sculptin is cleaved by factor Xa after each repeat domain at the LEARSDV motif, which correspond to P4, P3, P2, P1, P1′, P2 and P3′ subsite respectively. Factor Xa is highly specific for arginine at P1 position and glycine at P2 position^28^. However, in case of sculptin, factor Xa recognized alanine very well at P2 position. The effectiveness of hydrolysis and the verification that all fragments were cut in the same position showed it is not an unspecific degradation of sculptin by factor Xa. As far as we know, we reported here for the first time the specificity of factor Xa for alanine at position P2. The amino acid sequence LEAR can be found in many proteins including spectrin beta, centriolin, Ras-GTpase activating-like, myosin, and TP53 regulatory kinase, etc. These proteins might be chopped by factor Xa and may modulate its function particularly in tumor cells, where increased expression of factor Xa has been observed^29–33^. However, in-depth investigations are required to elucidate functional modulation of those proteins after cleavage by factor Xa. The amino acid sequence LEAR is present at the start of each repeated domain of sculptin. Factor Xa recognizes this sequence in each repeat and cleave it. Therefore, we observed different fragments including mono-, di- and tri-repeated domains of sculptin (Table 1). In addition, all those fragments of sculptin retained the thrombin inhibition activity but to a different extent. For instance, H1, H2, H3 and H4 fragments retained thrombin inhibition activity by approximately 50%, 45%, 70% and 80% respectively, compared to the intact sculptin molecule’s activity. The data suggest that the inhibition is not a simple sum of each domain because the molecules (H1 and H2) that have a single domain can retain 45–50% of total thrombin inhibition compared to the whole sculptin molecule. Likewise, two-repeated domains (H3) and three-repeated domains (H4) are not much different in terms of thrombin inhibition. Even a single domain of sculptin alone seems to have much higher thrombin inhibition potential than bivalirudin and argatroban^13, 24, 26, 27^. Computational analysis suggest that the single domain of sculptin, such as H1 and H2 fragments, may bind to thrombin in a fashion similar to hirudin and bivalirudin i.e. a bivalent thrombin inhibitor that occupies active site and exosite 1 (Fig. 7)^2, 8^. The comparative modelling and docking data proposed that Lys^23^ (at P1 position) of sculptin is more flexible and its side chain lodges in the active site pocket of thrombin, which makes it comparatively labile to thrombin hydrolysis. Furthermore, the predicted hydrogen bonds in the sculptin (single domain)-thrombin complex were equal to those in the C-terminal domain of hirudin-thrombin complex, thus indicating a somewhat similar affinity, which is in agreement with our kinetics data. The distance between Ser^195^ of thrombin and the peptide bond at P1 position (16.1 Å) for sculptin indicates that its binding may slowly change the conformation of the active site of thrombin. Then sculptin is hydrolyzed (*k* = 3.4 ± 0.7 × 10^−8^ M^−1^ h^−1^). In addition the residues at P1′, P1, P2 and P3 position for sculptin (^22^PKMP^22^) deviated in term of RMSD value from that of hirudin (^46^PKPQ^49^). We, therefore, expect a somewhat different mechanism for sculptin and hirudin inhibition of thrombin. In addition, the secondary structure was not predicted in sculptin, thus, we believe, it probably inhibits thrombin in trivalent manner i.e. it may bind to the active site, exosite 1 and exosite 2^8, 34^ (Fig. S7). However, X-ray crystallography studies are required to prove our hypothesis of a divalent nature for single domain and a trivalent nature for the whole sculptin. Furthermore, we propose that a single domain of sculptin and the whole sculptin could both become extremely effective antithrombotic drugs. For instance, as an antithrombotic agent of short duration, only a single domain of sculptin might be beneficial, while for a longer effective period di-, tri or tetra repeated domain forms of sculptin might be useful. Still, additional studies are required to investigate the effectiveness of mono-, di-, tri- domain and the whole sculptin in thrombosis. At last, we observed the effect of sculptin on global coagulation parameters. As anticipated, sculptin prolongs extrinsic (PT), intrinsic (aPTT) and common (TT) coagulation pathways in a concentration dependent manner because all of them use the common pathway, which ends up in the generation of fibrin by thrombin. However, the sensitivity of global coagulation parameters to sculptin are slightly different from each other. For instance, PT and aPTT require 12 and 25 nM, respectively, to reach the maximum time of the assay. On the other hand, the more sensitive TT requires 300 pM of sculptin to reach maximum limit. Nevertheless, sculptin prolonged all global coagulation parameters tested. Altogether, our results allow us to conclude that sculptin and its independent domains have strong potential to become a novel antithrombotic therapeutic.

## Materials and Methods

### Materials

All chemicals were purchased from Sigma-Aldrich, Merck or Fisher, unless otherwise indicated and were analytical grade or better. Human thrombin, factor Xa, plasmin and mass spectrometry grade trypsin (gold) were purchased from Promega (Madison, WI, USA). The chromogenic substrates S-2238 (H-D-Phe-Pip-Arg-pNA·2HCl) for thrombin, S-2765 (Z-D-Arg-Gly-Arg-pNA·2HCl) for factor Xa and S-2251 (H-D-Val-Leu-Lys-pNA·2HCl) for plasmin were purchased from Chromogenix (Chromogenix, UK). All solutions and buffers were prepared with Milli-Q water (Millipore, Germany).

### Phylogenetic analyses

Protein sequences of thrombin inhibitors were retrieved from Swiss-Prot/TrEMBL database (www.uniprot.org). The protein sequences were aligned using ClustalW command inbuilt in MEGA 7.0^35–37^. The phylogenetic profile was inferred using the Neighbor-Joining method with the bootstrap consensus from 100 replicates. The evolutionary distances were calculated using the p-distance method and all ambiguous positions were removed for each sequence pair^35, 38^.

### Cloning, expression and purification of recombinant sculptin

Sculptin full coding sequence was identified in the analysis of a cDNA library of the salivary glands from *Amblyomma cajennense* tick^19^. Codon optimization, gene synthesis and molecular cloning of sculptin into plasmid pET28a (Novagen/EMD Millipore, Germany) were conducted by GenOne (GenOne Inc. Brazil). Recombinant pET28a-Sculptin plasmid, which encodes the protein with a C-terminal histidine tag, was used to transform into chemically competent *E*. *coli* BL21 (DE3) strain. Transformed cells were inoculated in 500 mL of 2xYT culture medium (supplemented with 20 µg/mL Kanamycin) at 37 °C with 280-rpm agitation. Protein expression was induced at OD_600_ 0.6 by the addition of 1 mM IPTG and was further incubated for 4 h at 37 °C. For sculptin purification, cells were harvested, washed with 150 mM NaCl and re-suspended in lysis buffer^39^. After incubation for 1 h, cells suspension was sonicated (3 cycles of 1 min sonication at 70% potency with 1 min interval). The sample was centrifuged at 16000 rpm for 1 h at 4 °C. Sculptin was purified from the supernatant using a HisTrap HP (5 mL; GE Healthcare, USA) column of IMAC chromatography (AKTA AVANT; GE Healthcare, USA). Fractions containing the eluted protein were pooled and desalted using HiPrep 16/20 column (GE Healthcare, USA). The desalted sample was subjected to a CaptoQ ion-exchange column (GE Healthcare, USA). Fractions containing the purified protein were pooled and its buffer was exchanged to 50 mM phosphate buffer containing 150 mM NaCl, pH 7.4 using Amicon filters (3 kDa cut-off, Merck Millipore, Germany).

### Inhibition assays and kinetics of thrombin inhibition by sculptin

The inhibition of thrombin was assayed as described elsewhere^12^. Sculptin at different concentrations (0–5000 pM) was mixed with 15 µM S-2238 in 50 mM phosphate buffer containing 150 mM NaCl and 0.1% PEG 6000, pH 7.4 at 37 °C. Subsequently, 100 pM thrombin was added to each reaction mixture and the hydrolysis of S-2238 was monitored photometrically for 60 min at 405 nm (SpectraMax 190, Molecular Devices, USA). The percentage of inhibition was determined as described elsewhere^14^. For IC_50_ calculation, the percent inhibition of thrombin was plotted versus the log of sculptin concentration using equation 1 of dose-response function.

1 $${\rm{y}}={\rm{A}}1+\frac{{\rm{A}}2-{\rm{A}}1}{1+{10}^{({\rm{LOGx}}0-{\rm{x}})\ast {\rm{p}}}}$$

The rate of formation of p-nitroaniline was determined using the extinction coefficient of p-nitroaniline (8270 M^−1^ cm^−1^). For Hanes–Woolf plot, the Michaelis-Menten equation was rearranged to get equation 2, substrate concentration divided by initial velocity was plotted with substrate concentration. The inhibition constant Ki was calculated using equations 3 and 4, where, Km is Michaelis-Menten constant (without inhibitor), apparent Km is Km in the presence of inhibitor, Vmax is maximal velocity, and K_i_ is the inhibition constant and is in the same unit as of inhibitor.

2 $$\frac{[S]}{V}=\frac{1}{Vmax}\,[S]+\frac{Km}{Vmax}$$

3 $${\rm{apparent}}\,{\rm{Km}}=\mathrm{Km}\,(1+\frac{[{\rm{I}}]}{{\rm{Ki}}})$$

4 $${\rm{Y}}={\rm{Vmax}}\ast {\rm{X}}/({\rm{KmObs}}+{\rm{X}})$$

In addition, dose-response curve was fitted by non-linear regression using GraphPad Prism 5.0 (GraphPad Software, CA, USA). Fractional velocities were plotted versus inhibitor concentrations using Morrison’s tight binding equation 5, where Vi and V0 is the steady state in the presence of inhibitor and the uninhibited velocities respectively. [E] is the enzyme concentration and [I] is the inhibitor concentration. K_i_ app is the apparent inhibition constant. To obtain observed *k*, the progress curves were analyzed by the time dependent inhibition equation 6, where [P] is the concentration of p-nitroaniline released from S-2238 hydrolysis, vi and vs are the initial and steady state velocities, respectively, in the presence of inhibitor, and *k*_obs_ is the apparent first order rate constant. The apparent first-order rate constants were plotted as function of sculptin concentration, where the intercept of the plot is *k*_on_ and its slope is *k*_off_. The inhibition constant K_i_ can be calculated from equation 7.

5 $$Vi/V0=1-([E]+[I]+Ki\,app-\sqrt{([E]+[I]+Ki\,app)2-4[E][I])})/2[E]$$

6 $$[{\rm{P}}]={\rm{Vs}}\,{\rm{t}}+{\rm{Vi}}-\frac{{\rm{Vs}}}{{\rm{kobs}}}\,[1-\exp (-{\rm{kobs}}\,{\rm{t}})]$$

7 $${\rm{Ki}}=\frac{{\rm{koff}}}{{\rm{kon}}}$$

### Incubation of sculptin with serine proteases

Sculptin (10 µM) was incubated with 1 µM thrombin, 1 µM trypsin, 1 µM factor Xa and 50 µM phospholipids, or 1 µM plasmin in 50 mM phosphate buffer containing 150 mM NaCl and 0.1% PEG 6000, pH 7.4^40^. The reaction mixtures were incubated for 6 h or 18 h at 37 °C. The reaction mixtures were separated by 15% SDS-PAGE^39^. When required, reaction mixtures were subjected to MALDI-TOF mass spectrometry (Waters Corporation, USA).

### Thrombin inhibition assay of enzyme hydrolyzed sculptin

Sculptin (10 µM) was incubated without or with 1 µM of serine proteases (thrombin, plasmin, trypsin or factor Xa) in 50 mM phosphate buffer containing 150 mM NaCl and 0.1% PEG 6000, pH 7.4 at 37 °C. The reaction mixtures (20 µl) were separated by SDS-PAGE. After incubation of sculptin with serine protease (6 and 18 h), the reaction mixtures containing 10 µM sculptin were diluted (100 thousand times) to 100 pM final concentration. Each diluent was supplemented with 100 pM thrombin and subsequently added S-2238 chromogenic substrate. Hydrolysis of S-2238 by thrombin was monitored photometrically for 60 min at 405 nm (SpectraMax 190, Molecular Devices, USA).

### Comparative structure and docking of sculptin

Next, we conducted a comparative structure for the sculptin single domain using hirudin complex with thrombin (PDB id, 4 MLF) using Modeler software^41, 42^. The best comparative model for sculptin was selected on the bases of PROSA and PROCHECK energy profiles^42^. The comparative model was then subjected to the protein-protein docking using an automated Z-DOC^43^ and PlusPro^44^ software. Both software provided comparable docking results. The sculptin model docked into thrombin active site with the lowest energy was selected for further studies. For assurance, we also subjected the crystal structure of hirudin (from PDB id 4MLF) to the protein-protein docking using above software. The best docking of hirudin in the thrombin active site was obtained with zero RMSD. The RMSD value was further confirmed by SuperPose (http://wishart.biology.ualberta.ca/SuperPose/). The structures were visualized in discovery studio visualizer (http://accelrys.com).

### Global blood coagulation assays

The experimental procedures were conducted in agreement with the guidelines for the care and use of laboratory subjects of the Butantan Institute. The Ethics Committee for Research, Butantan Institute (CEP permit number 518.693) approved the study on human blood. Informed consent was obtained from the volunteers conferring to the declaration of Helsinki Convention and the Brazilian Department of Health. Blood was collected from healthy human volunteers and plasma was isolated immediately by centrifugation of blood at 1000 g for 15 min. The plasma were supplemented with different concentration of sculptin and activated partial thromboplastin time (aPTT), prothrombin time (PT) and thrombin time (TT) assays were conducted using commercially available kits, as described elsewhere^14^.

### Edman degradation

Sculptin (10 µM) was incubated with 1 µM thrombin or 1 µM factor Xa and 50 µM phospholipids in 50 mM phosphate buffer containing 150 mM NaCl and 0.1% PEG 6000, pH 7.4. The products resulting from the sculptin digestion with either factor Xa or thrombin were separated using a Phenomenex 250 × 4.6 mm C-18 Jupiter column (Phenomenex, USA) connected with a Shimadzu LC-20A binary HPLC (Shimadzu, Japan). The peaks were collected and were dried. N-terminal residues of each peak were sequenced on a PPSQ21A protein sequencer (Shimadzu, Japan) according to the manufacturer’s instructions^45^.

### Mass spectrometry analysis of the sculptin incubated with serine protease

The samples obtained from sculptin incubation with factor Xa or thrombin were analyzed by MALDI-TOF MS, using 3,5-dimethoxy-4-hydroxycinnamic acid as the matrix on AXIMA Performance MALDI TOF/TOF Mass Spectrometer (Shimadzu, Japan)^39^. Briefly, the matrix solution (1 µL) and sculptin digest (1 µL) of either factor Xa or thrombin were premixed in eppendorf tube and 0.5 µL of the pre-mixed sample was spotted on the steel target plate. The mass spectra were recorded by MALDI-TOF instrument (Shimadzu, Japan). A linear time-of-flight in MALDI-MS instrument was employed with vacuum 3 × 10^−7^ Torr and power of laser 120. The spectra were generated by averages of 50–100 automatic laser shots. The mass spectra were analyzed using Axima Performance proteomics suit. The mass tolerance was 0.2%.

### Statistical analysis

Data are expressed as the mean ± standard deviation and statistical significance was calculated for at least three independent experiments employing the one-way ANOVA using GraphPad Prism 5.0 software (GraphPad Software, Inc., USA) or OriginPro 8 (OriginLab Corp. USA). Values of p < 0.05 were considered significant.

## Electronic supplementary material

Supplementary data
