# Supplementary material for: Revisiting antithrombotic therapeutics; sculptin, a novel specific, competitive, reversible, scissile and tight binding inhibitor of thrombin
Source: Sci Rep. 2017 May 3;7:1431. doi: 10.1038/s41598-017-01486-w (PMC5431157; doi:10.1038/s41598-017-01486-w)
Supplement: Supplementary file 1 — Supplementary data [file 41598_2017_1486_MOESM1_ESM.pdf]

Revisiting antithrombotic therapeutics; sculptin, a novel specific, competitive, reversible, scissile and tight binding inhibitor of thrombin

Asif Iqbal[ab1], Mauricio Barbugiani Goldfeder[ab1], Rafael Marques-Porto[a], Huma Asif[c], Jean Gabriel de Souza[ab], Fernanda Faria[ab] and Ana Marisa Chudzinski-Tavassi[ab]*

[a] Laboratory of Biochemistry and Biophysics, Butantan Institute, Sao Paulo, SP, Brazil.

[b] Centre of Excellence in New Target Discovery (CENTD), Butantan Institute, São Paulo, SP, Brazil

[c] Laboratory of Gene Expression in Eukaryotes, Butantan Institute, São Paulo, SP, Brazil

*Corresponding author:

Biochemistry and Biophysics Laboratory, Butantan Institute Av. Vital Brazil, 1500 - CEP 05503-900 - São Paulo, SP, Brazil. Phone: 551126279738; Fax: 551126279505.

Email: ana.chudzinski@butantan.gov.br

[1]The authors contributed equally to this work

A

```
         10         20         30   *     40   #     50         60
MEKNIIVLLA VALTGPAVLA KPQGHPHDAL EARSDAVHTA VPKMPKGGHG GFEPIPIDYD

      *  70      #  80         90        100 *      110 #      120
ERALEARSDA VHTAVPKMPK GGLGGFEPIP IDYDERALEA RSDAVHTAVP KMPKGGHGGF

        130   *   140     #   150        160
EPIPIDYDER ALEARSDAVH TAVPKMPKGG HGGFEPIPID YDERALHA
```

B

```
Acajennense        ---------------------KPRGYP--HDALEARSDAVH-TAVPKMPK--GGHGGFEP
Sculptin           --------------------AKPQGHP--HDALEARSDAVH-TAVPKMPK--GGHGGFEP
Rappendiculatus    MPRQNLRQRKLSNDLEARAAVASTDYESDEDSVGGGSKGSA-VARPKAKRDNSGSGDFES
Hmedicinalis       VVYTDCTESGQNLCLCEGSNVCGQGNKCILGSDGEKNQCVTGEGTPKPQS--HNDGDFEE

Acajennense        IPID-YEER--
Sculptin           IPID-YDER--
Rappendiculatus    IPIPGMRDSQA
Hmedicinalis       IPEE-YLQ---
```

Fig. S1. Sequence analysis of sculptin. (A) Sequence of sculptin identified in the transcriptomic analysis of the tick salivary glands. The predicted signal peptide is underlined. The four times-repeated peptide within sculptin is shown in alternating gray and yellow colors. (B) Multiple alignment of sculptin with hirudin from *Amblyomma cajennense*, *Rhipicephalus appendiculatus* and *Hirudo medicinalis*. The conserved residues are highlighted in gray. The active site binding P of hirudin from *Hirudo medicinalis* is changed to M in sculptin (highlighted in yellow). Factor Xa and thrombin cleavage sites are identified by an asterisk and hashtag respectively.

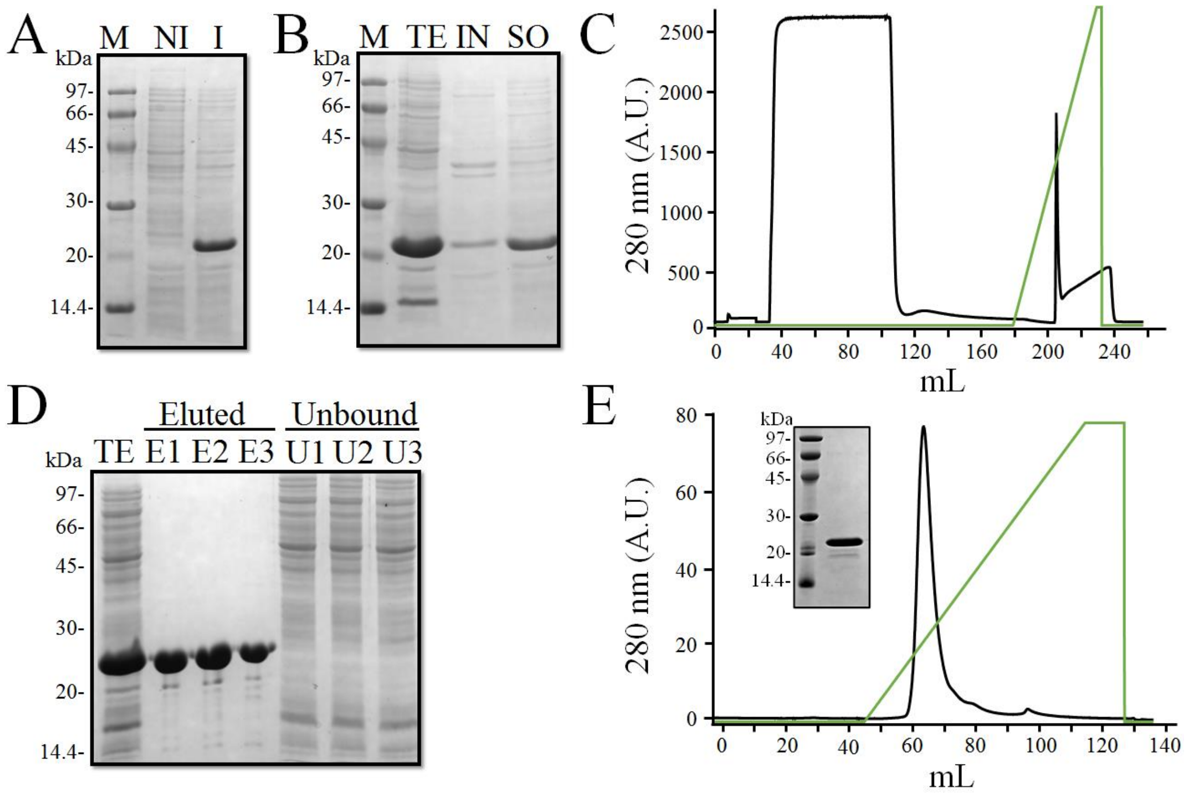

Fig. S2. Expression and purification of sculptin. Sculptin synthetic gene was cloned into the pET28a expression vector and the recombinant protein was expressed in *E. coli* BL21 (DE3) in liquid medium at 37°C. Whole cell lysates of non-induced or induced (IPTG 0.5 mM) cultures were analyzed by SDS-PAGE (15 %). (A) SDS-PAGE of recombinant sculptin induction. Lanes M, NI and I represent protein marker, not induced and induced with IPTG, respectively. (B) SDS-PAGE of the cell lysate of *E. coli* after expression of sculptin. Lanes M, TE, IN, SO correspond to protein marker, total extract, insoluble and soluble fractions respectively. (C) Affinity chromatography purification of sculptin. The soluble fraction was filtered through a 0.45μm membrane and was applied on a His-tag Ni-chelating affinity column. Bound protein was eluted with imidazole and 15μl of each fraction was analyzed by SDS-PAGE. (D) SDS-PAGE of fractions from affinity chromatography. Lanes TE, E, and U represent total extract, eluted protein fractions and unbound protein fraction respectively. (E) Ion-exchange chromatogram of sculptin purification. Inset showing the SDS-PAGE of the purified sculptin band.

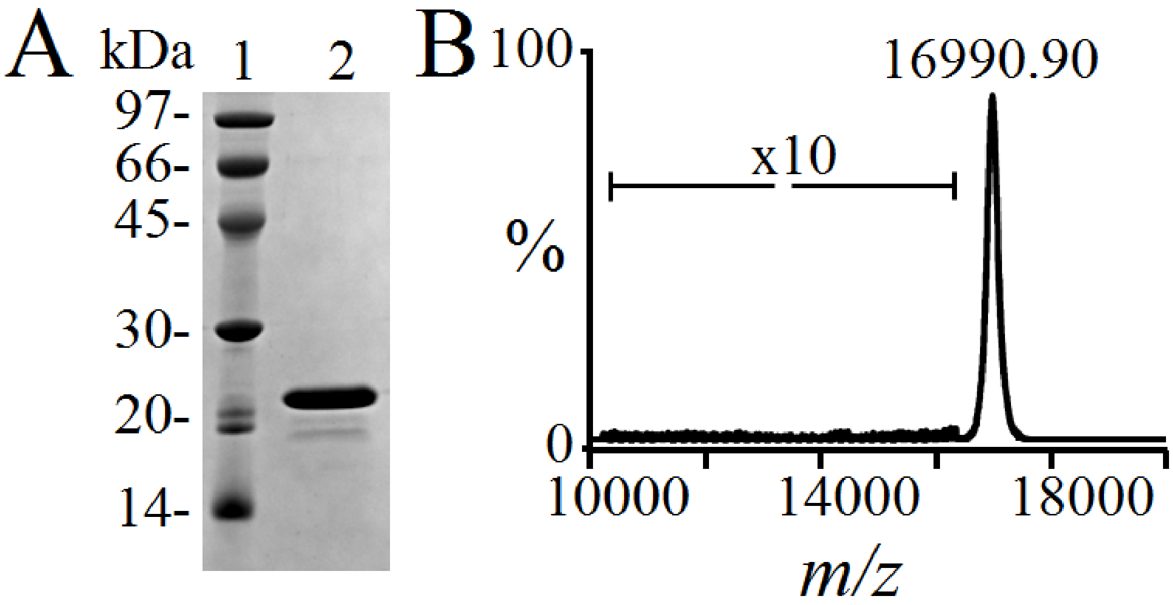

Fig. S3. SDS-PAGE and MALDI mass spectrometry of purified sculptin. (A) SDS-PAGE of purified sculptin using conventional chromatographic methods (see experimental procedures and figure S2). (B) MALDI-TOF MS spectrum of purified sculptin.

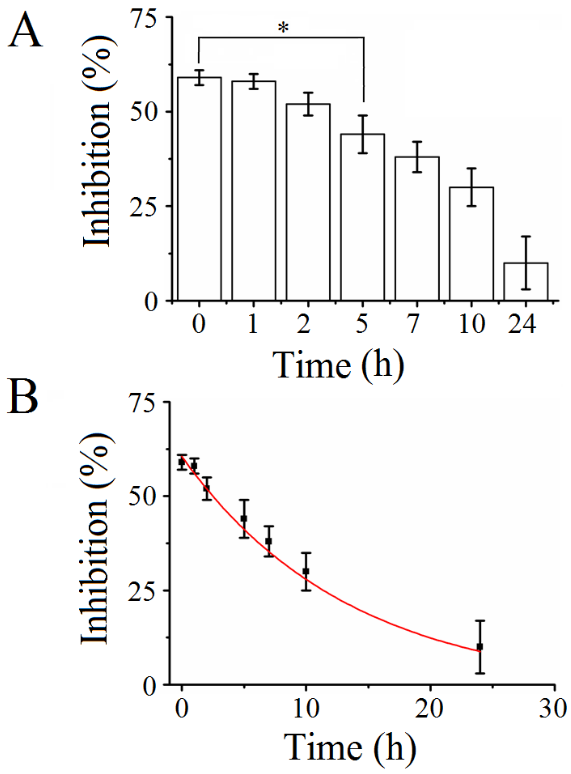

Fig. S4. Kinetics of sculptin deactivation by thrombin. Thrombin (1 µM) was incubated with or not sculptin (10 µM) in 50 mM phosphate buffer containing 150 mM NaCl and 0.1% PEG 6000, pH 7.4 at 37°C for different time. The reaction mixture at different time intervals was diluted to 100pM final concentration of sculptin and then supplemented with 0.1 nM fresh Thrombin. The hydrolysis of the S-2238 chromogenic substrate by thrombin in the presence and absence of sculptin was monitored by spectrophotometry. (A) The plot of the inactivation of sculptin with time. (B) The plot of the inactivation of sculptin with time fitted in exponential decay. The red line showing the single exponential fit. The experimental condition of (B) is the same as (A).

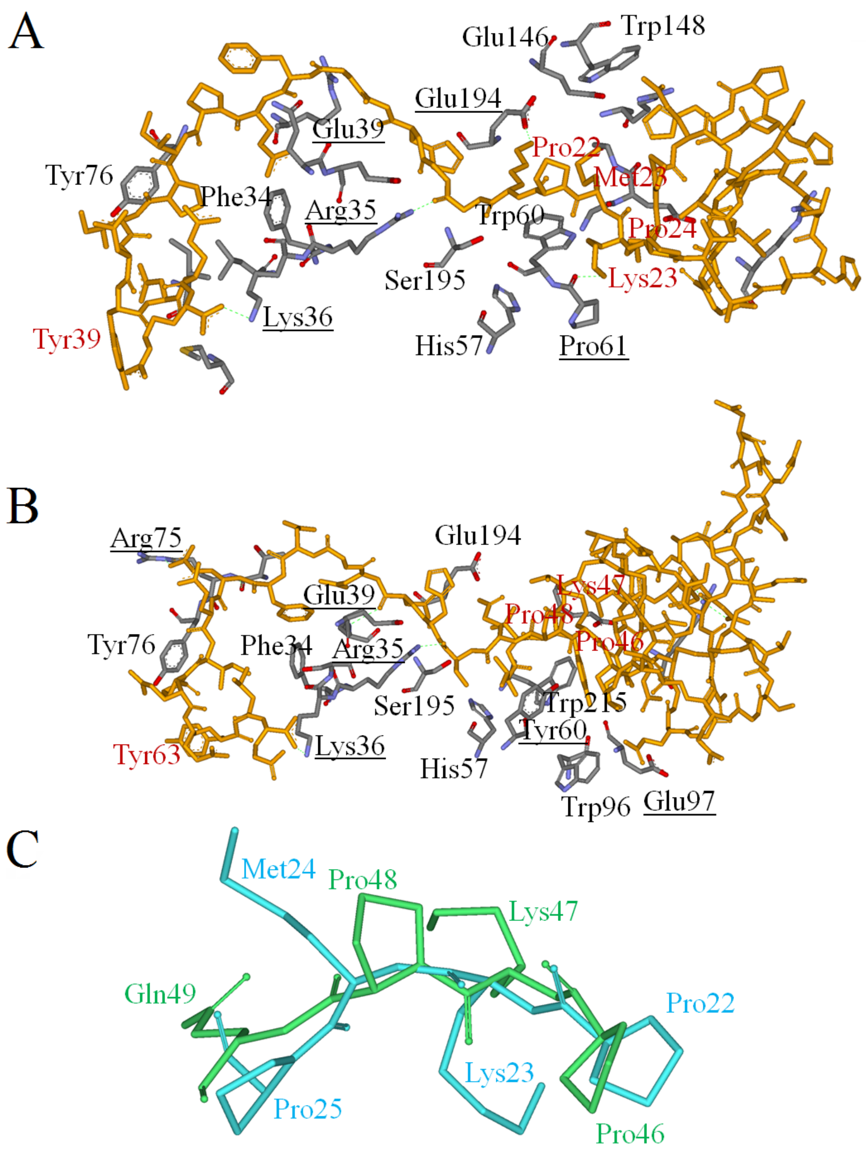

Fig. S5. Cartoon (stick) representation of the interaction of sculptin (single domain) and hirudin with the active site residues of thrombin. Docking of the comparative model of the single domain of sculptin into the thrombin active site was accomplished as discussed in the experimental procedures. (A) Interaction of the single domain of sculptin with residues inside active site pocket of thrombin. (B) Interaction of hirudin with residues inside active site pocket of thrombin. The inhibitor (sculptin/hirudin) is shown in yellow and thrombin residues in elemental gray. (C) The superposition of residues at P1`, P1, P2 and P3 position of hirudin (^{46}PKPQ^{49}; cyan) and sculptin (^{22}PKMP^{22}; green). The residues of the inhibitor forming hydrogen bonds with thrombin residues are underlined. The residue number is for sculptin without signal peptide.

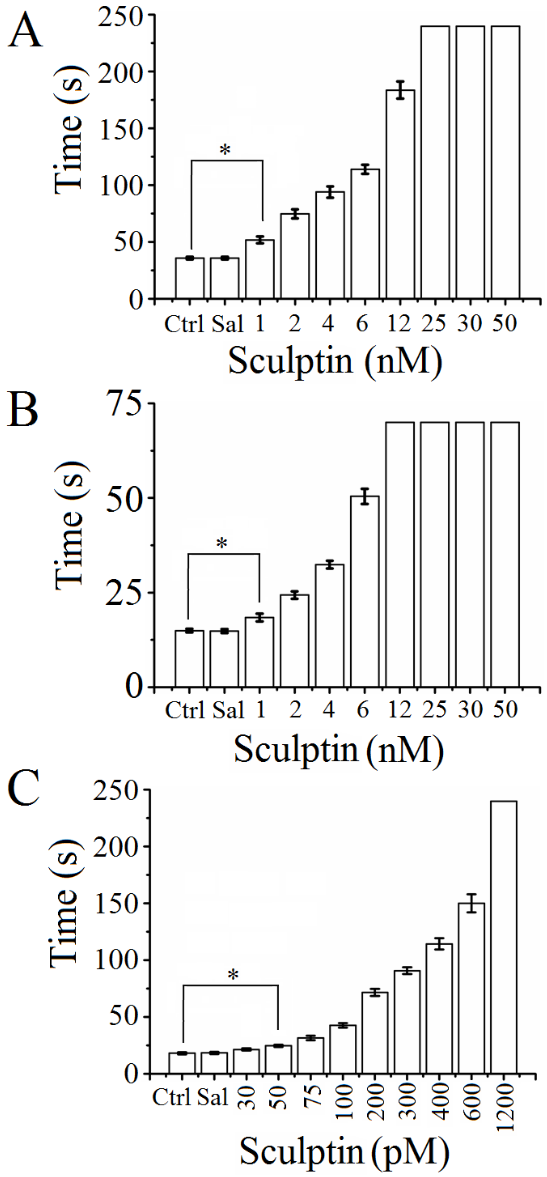

Fig. S6. In vitro, aPTT, PT and TT evaluation in isolated human plasma incubated with different concentrations of sculptin. Plasma was obtained from the blood of healthy human volunteers and incubated with different concentrations of sculptin. APTT, PT and TT were determined as in experimental procedure. (A) Activated partial thromboplastin time (B) prothrombin time and (C) thrombin time. Ctrl refers to plasma and Sal refers to plasma plus saline solution. The results shown in (A), (B) and (C) correspond to the mean ± standard deviation values acquired in three independent experiments; $*p < 0.05$.

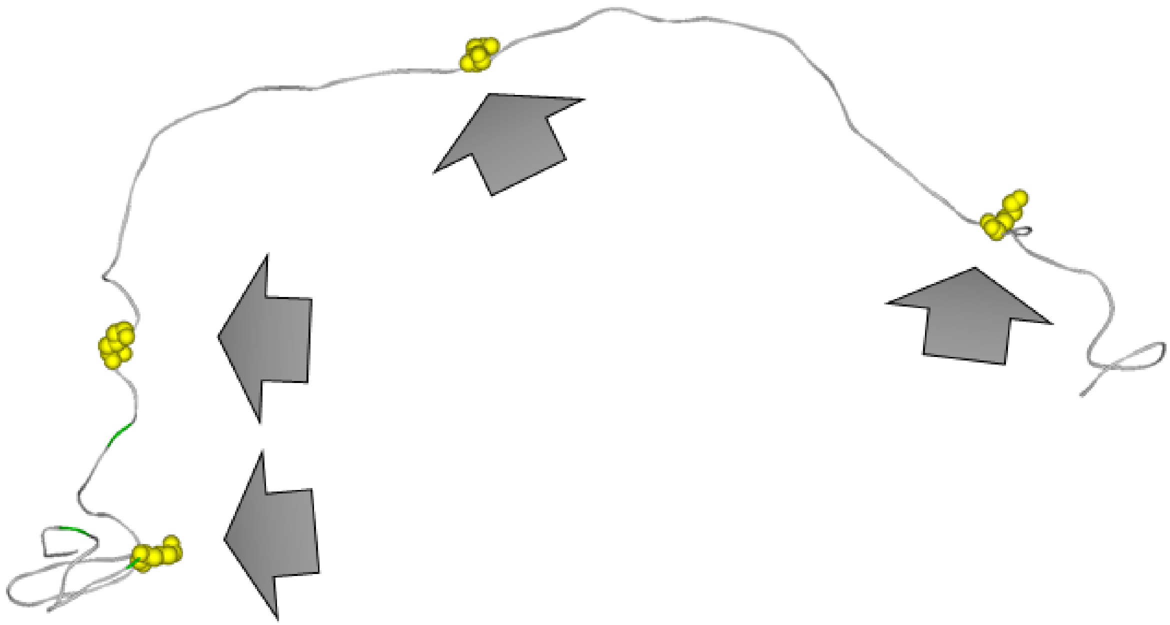

Fig. S7. Cartoon (Solid ribbon) representation of sculptin. There is enough room for thrombin enzyme to accommodate in sculptin. In other words, sculptin may surround thrombin and inhibit it in trivalent manner i.e. block active site, exosite-1 and exosite-2. Lys residue of the inhibitors is shown in yellow and the arrows indicate that any of these positions may occupy the thrombin active site.

Table S1. The fragments of sculptin generated by thrombin. Sculptin (10 µM) was incubated with 1 µM of thrombin in 50 mM phosphate buffer containing 150 mM NaCl and 0.1% PEG 6000 pH 7.4 for 4 h at 37°C. The reaction mixtures were separated by reversed phase C-18 HPLC column. The fractions were subjected to Edman sequencing and MALDI-TOF mass spectrometry.

Peak number [a]	Fragment of sculptin [b]	Theoretical Mass [MH]+	Calculated Mass [c] [MH]+
**1**	**GKPQG**HPHDALEARSDAVHTAVPK	2518.77	2521.74
	**GKPQG**HPHDALEARSDAVHTAVPKMPKGGHGGFEPIPIDYDERALEARSDAVHTAVPK	6162.85	6169.56
**2**	**MPKGG**HGGFEPIPIDYDERALEARSDAVHTAVPK	3663.09	3663.90
**3**	**MPKGG**HGGFEPIPIDYDERALHALEHHHHHH	3572.92	3572.70
**5**	**MPKGG**HGGFEPIPIDYDERALEARSDAVHTAVPKMPKGGHGGFEPIPIDYDERALHALEHHHHHH	7217.00	7202.66
**6**	**MPKGG**HGGFEPIPIDYDERALEARSDAVHTAVPKMPKGGLGGFEPIPIDYDERALEARSDAVHTAVPK	7282.19	7261.19
	**MPKGG**LGGFEPIPIDYDERALEARSDAVHTAVPKMPKGGHGGFEPIPIDYDERALEARSDAVHTAVPKMPKGGHGGFEPIPIDYDERALHALEHHHHHH	10837.10	10807.97
	**MPKGG**HGGFEPIPIDYDERALEARSDAVHTAVPKMPKGGLGGFEPIPIDYDERALEARSDAVHTAVPKMPKGGHGGFEPIPIDYDERALEARSDAVHTAVPKMPKGGHGGFEPIPIDYDERALHALEHHHHHH	14481.18	14431.37
	**GKPQG**HPHDALEARSDAVHTAVPKMPKGGHGGFEPIPIDYDERALEARSDAVHTAVPKMPKGGLGGFEPIPIDYDERALEARSDAVHTAVPKMPKGGHGGFEPIPIDYDERALEARSDAVHTAVPKMPKGGHGGFEPIPIDYDERALHALEHHHHHH	16981.94	16990.90

[a] Peak number of the HPLC chromatogram in Figure 5E (main text)

[b] Fragment of sculptin hydrolyzed by thrombin. The first 5 amino acids (bold) were sequence by Edman degradation.

[c] The experimental average mass was calculated by MALDI-TOF mass spectrometry.

Table S2. The fragments of sculptin generated by factor Xa. Sculptin (10 µM) was incubated with 1 µM of factor Xa in 50 mM phosphate buffer containing 150 mM NaCl and 50 µM PS/PC pH 7.4 for 4 h at 37°C. The reaction mixtures were separated by reversed phase C-18 HPLC column. The fractions were subjected to Edman sequencing and MALDI-TOF mass spectrometry.

Peak number	Fragmentation of sculptin	Theoretical Mass	Calculated Mass
**1**	**GKPQG**HPHDALEARSDAVHTAVPKMPKGGHGGFEPIPIDYDERALEAR	5156.74	5153.57
**2**	**SDAVH**TAVPKMPKGGHGGFEPIPIDYDERALEAR	3663.10	3667.50
	**SDAVH**TAVPKMPKGGHGGFEPIPIDYDERALHALEHHHHHH	4579.05	4582.40
**3**	**SDAVH**TAVPKMPKGGHGGFEPIPIDYDERALEARSDAVHTAVPKMPKGGHGGFEPIPIDYDERALHALEHHHHHH	8223.13	8220.55
	**SDAVH**TAVPKMPKGGHGGFEPIPIDYDERALEARSDAVHTAVPKMPKGGHGGFEPIPIDYDER	6765.55	6770.60
**4**	**SDAVH**TAVPKMPKGGHGGFEPIPIDYDERALEARSDAVHTAVPKMPKGGHGGFEPIPIDYDERALEAR	7306.17	7299.61
	**GKPQG**HPHDALEARSDAVHTAVPKMPKGGHGGFEPIPIDYDERALEARSDAVHTAVPKMPKGGLGGFEPIPIDYDERALEARSDAVHTAVPKMPKGGHGGFEPIPIDYDERALEAR	12420.90	12427.54
	**GKPQG**HPHDALEARSDAVHTAVPKMPKGGHGGFEPIPIDYDERALEARSDAVHTAVPKMPKGGLGGFEPIPIDYDERALEARSDAVHTAVPKMPKGGHGGFEPIPIDYDERALEARSDAVHTAVPKMPKGGHGGFEPIPIDYDERALHALEHHHHHH	16981.94	16990.90

[a] Peak number of the HPLC chromatogram in Figure 5F (main text)

[b] Fragment of sculptin hydrolyzed by factor Xa. The first five amino acids (bold) were sequence by Edman degradation.

[c] The experimental average mass was calculated by MALDI-TOF mass spectrometry.

Table S3. Comparison of biochemical properties of thrombin inhibitors.

Inhibitor [a]	Inhibition type	K_i value	Administration	Half-life [b]	Refs
**Recombinant hirudin[d]**	Competitive	19 ± 2 pM	Intravenous	1.3 h	[11,12]
**Sulfo-hirudin[d]**	Competitive	1.2 ± 0.2 pM	ND	ND	[27,46]
**Hirugen[d]**	Non-competitive	1.3 ± 0.2 µM	ND	ND	[25]
**Bivalirudin[d]**	Non-competitive	1.9 ± 2.6 nM	Intravenous	25 min	[13,26,27]
**Argatroban**	Non-competitive	39 ± 2 nM	Intravenous	50 min	[24]
**Sculptin[e]**	competitive	18.5 ± 2.2 pM	ND	ND<br>8 h [c]	This study

[a] Direct thrombin inhibitors

[b] Half-life in plasma of human healthy volunteers.

[c] Half-life in plasma ex-vivo and in 50 mM phosphate buffer containing, 1 µM thrombin, 10 µM sculptin and 150 mM NaCl and 0.1% PEG 6000 pH 7.4 for 4 h at 37°C.

ND, not determined

[d] Bivalent thrombin inhibitor, which occupies active site and exosite 1.

[e] Proposed to be bivalent (single domain) or trivalent (intact molecule)

Table S4. Comparison of binding kinetics sculptin with hirudin from leech.

Inhibitor	k_{on} $\times 10^{7}$ $M^{-1}s^{-1}$	k_{off} $\times 10^{-3}$ s^{-1}	$K_i = k_{off}/k_{on}$ $\times 10^{-12}$ M
**Natural hirudin**	30.8 ± 0.2	0.9 ± 0.4	3 ± 1[1]
**r-Hirudin**	7.8 ± 0.2	1.5 ± 0.2	19 ± 2[1]
**Sculptin**	4.04 ± 0.03	0.65 ± 0.04	16.1 ± 1.4 This study

References

1. Dodt, J., Köhler, S. & Baici, A. Interaction of site specific hirudin variants with α-thrombin. *FEBS Lett.* **229,** 87–90 (1988).
